# Development of working memory and inhibitory control in early childhood: Cross-sectional analysis by age intervals and gender in Ecuadorian preschoolers

**DOI:** 10.1371/journal.pone.0299394

**Published:** 2024-05-14

**Authors:** Sofía López-Vallejo, Carlos Burneo-Garcés, Miguel Pérez-García

**Affiliations:** 1 Department of Personality, Assessment, and Psychological Treatment, University of Granada (UGR), Granada, Spain; 2 The Brain, Mind, and Behavior Research Center at the University of Granada (CIMCYC-UGR), Granada, Spain; 3 University of Otavalo, Dirección de Posgrado, Otavalo, Ecuador; 4 Escuela de Psicología, Universidad de las Américas, Quito, Ecuador; University of St Andrews, UNITED KINGDOM

## Abstract

Working memory (WM) and inhibitory control (IC) play a crucial role in learning during early childhood. The literature suggests a non-linear developmental trajectory of executive functions (EFs) with varied results according to gender, usually attributed to environmental factors. However, there is insufficient and inconclusive data on whether this pattern is reproduced in the Latin American preschool population since most studies have been conducted in English-speaking, European, and Asian environments. Thus, objectively comparing children’s executive performance across diverse international geographical contexts becomes challenging. This study aimed to conduct a cross-sectional analysis of the performance in WM and IC of 982 Ecuadorian preschoolers aged between 42 and 65 months (*M* = 53.71; *SD* = 5.714) and belonging to medium-high, medium, and low-medium socioeconomic strata. The participants consisted of 496 boys (*M* = 53.77; *SD* = 5.598) and 486 girls (*M* = 53.65; *SD* = 5.834), representing nine cities in Ecuador. To assess the effect of age and gender on performance in these two domains, the sample was divided into four 6-month age intervals. Two tests were administered to the participants, and a survey was conducted with 799 of their usual caregivers. Viewing the cross-sectional mean scores of the WM and IC tests as a temporal continuum reveals an upward trend in each age interval studied. Girls outperformed boys on the IC test, showing statistically significant differences in the earliest age interval. The gender differences in executive performance reported in the literature emphasize the need to explore the modulating effect of environmental variables on early childhood development. This information could offer valuable insights for adapting and optimizing cognitive and didactic strategies in early childhood tailored to the characteristics and needs of the preschool population.

## Introduction

Neurodevelopment involves a complex interaction between genetic and environmental factors, with critical stages and changes occurring during the prenatal period and the initial years of life [1]. These changes, stemming from brain maturation, are manifest in several domains, including cognition [2]. Within this developmental process, executive functions (EFs) play a modulating role in the generation, execution, regulation, readjustment, and monitoring of behaviors to achieve complex goals, particularly those that require a novel and creative approach [3]. The significance of these functions explains the interest in exploring their developmental trajectory in early childhood. The existing literature indicates non-linear trajectories of EF development, although most studies on this topic have been conducted in English-speaking [3, 4], European [5, 6], and Asian contexts [7, 8]. It is important to note that the non-linear trajectory of a measure should be understood as the non-identical relationship between mean scores at different moments or age intervals analyzed as a temporal continuum. Given the emerging nature of research in this field within Latin American populations [9–11], it remains to be seen whether this observed pattern is replicated in a socio-cultural context distinct from those mentioned above. If we add to this the size of the Latin American child population in terms of global statistics [12], approaching the study of EFs in this context assumes considerable importance.

According to Diamond’s EFs model, Working Memory (WM), Inhibitory Control (IC), and Cognitive Flexibility (CF) play pivotal roles as they underlie all executive processes during this developmental stage [13, 14]. Additionally, these three domains form the basis of other higher-order processes such as decision-making, problem-solving, and planning [3]. Diamond’s model is particularly noteworthy since it organizes the EFs according to their degree of maturation and functional interrelation in early childhood [13, 15]. Therefore, in this unitary conception of EFs, this author assigns a leading role to WM and IC, considering their maturation as a determining factor in the development of CF and higher-order EFs [15, 16]. WM is involved in the retention and storage of information essential for completing a task, engaging in simultaneous activities, or following complex instructions, while IC is essential for regulating the inclination to perform habitual behaviors or those linked to immediate satisfaction [17–19]. Notably, both WM and IC, distinguishable from the age of 2 and 3 years, respectively, significantly impact the learning process [20, 21], where their degree of maturation serves as a predictor of academic performance, behavioral adjustment, social competencies, and resilience [22, 23]. Thus, the two-factor model of EFs (IC and WM) in early childhood [6, 24, 25] is consistent with the dynamics of cortical connection processes [26–29], axonal myelination [30–32], and changes in cortical thickness, brain volume, and activation patterns in frontoparietal and frontostriatal neural circuits [33].

Given the above, exploring the developmental trajectory of EFs can be considered a priority from any study approach. This knowledge is crucial to understanding the functional developmental parameters of any population and context, particularly in such a sensitive stage of brain maturation and plasticity as early childhood [30]. In this regard, the strategic organization and application of teaching-learning strategies aligned with the accurate timing of functional maturation and networking of EFs hold the potential to achieve improved results [4, 14, 34]. Moreover, research in this area gains further relevance when considering that the development of WM and IC during early childhood predicts better performance in reading, mathematics, and science [8, 24, 35, 36].

As mentioned above, the literature on EF development—including WM and IC—in preschoolers from English-speaking, European, and Asian populations suggests a non-linear trajectory [3–8, 19, 37]. The most relevant findings are reviewed below.

In an American population, 17 instruments were used to assess EFs in a sample of 602 preschoolers aged 2 to 6 years, aiming to identify the most age-sensitive measures and the difficulty level of each task across age intervals (2–3, 3–4, 4–5, and 5–6 years). Of the 17 tests applied, 11 detected a non-linear improvement in EFs in the age intervals studied [26]. A broader examination of attention and EFs involving 243 preschoolers aged between 2 and 6 years revealed an age-related development of EFs, with older children outperforming their younger counterparts. The focus on IC in this study highlighted changes attributable to an enhanced stimulus-response relationship [4]. They used a final sample ranging from 166 to 188 children for each measure, aged 3 to 15 years, divided into two age intervals: 3 to 6 and 8 to 15 years. The results revealed age-related cognitive changes during the child’s neurodevelopment, explained by fundamental changes to the structure of cognition [23]. Finally, a longitudinal study with Australian children aged 5 to 7 reported an improvement in IC over time [34]. Similar results were observed in Spanish preschoolers [38], as well as in British and Korean preschoolers [29, 39] aged 3 to 6 years in both cases.

Comparing EFs among child populations from different geographic contexts has gained considerable attention from researchers, recognizing that the trajectory of EFs may show specific characteristics depending on the socio-cultural environment. For instance, a study involving children aged 9 to 16 from Hong Kong (HK) and the United Kingdom (UK) revealed similar age-related improvements, although HK children outperformed their UK counterparts in all EF tasks. The average EFs score for HK children at 10 and 12 years matched that of UK children at 12 and 14 years, respectively [37, 38]. This difference was also evident in preschoolers from HK, who performed better on EFs and theory of mind tasks compared to those from the UK [7]. Similar research demonstrated that Korean 3-year-olds outperformed their nearly 5-year-old English counterparts on inhibition tasks, although no statistical differences in WM scores were identified [40]. Comparisons between Asian and American children do not differ from previous findings. Thus, Japanese children showed significantly higher contextual sensitivity, which increased with age, particularly in set-shifting EF tasks compared to American children [8]. Furthermore, Chinese children aged 3–5 years also outperformed American children in inhibition and attentional control tasks [41] and EFs such as WM and IC [42]. While these studies suggest non-linear developmental trajectories of EFs, they also acknowledge, to varying degrees, that the results may differ according to the socio-cultural environment. Unfortunately, the lack of such data in Latin American socio-cultural environments hinders the identification of similarities with findings from other studies. Accordingly, it is reasonable and of value to investigate the characteristics of the developmental trajectory of EFs in populations exposed to specific and diverse living conditions [12, 43].

Regarding gender, some authors have found differences in neuropsychological performance in early childhood, giving rise to an ongoing debate that remains unresolved. Notably, in both East and West Asian samples, girls have been reported to outperform boys on planning performance [44], behavioral regulation [45], IC, WM, and CF [46]. These results contrast with the findings of a meta-analysis that included data from both Western and Eastern countries, indicating the opposite trend—better WM performance in boys than girls [47]. Similarly, another study conducted in Tanzania reported that boys performed better than their female counterparts on direct EF tasks, with small effect sizes [48].

Several initial questions arise from the growing body of research showing differences in EFs (particularly WM and IC) among preschoolers from different geographical settings. These differences are typically attributed to environmental/cultural variables [32, 49, 50]. While the abundance of cross-cultural studies from affluent Asian, European, and English-speaking regions provides valuable insights, they do not represent the full spectrum of cultural and socioeconomic diversity at an international level [51, 52]. For instance, there is a scarcity of studies in Latin American populations, despite the pressing need to understand and analyze the determinants of neurodevelopment in early childhood given the cultural and socioeconomic diversity in the region [9, 10, 11, 51]. To our knowledge, there has been a notable absence of studies examining executive performance in early childhood according to age and gender within countries characterized by significant cultural and geographic diversity, such as Ecuador. With a population of 17 million people, Ecuador is geographically divided into four regions: Costa: 53.3%; Sierra: 41%; Amazon: 5.5%; and the Galápagos Islands: 0.2%. These regions encompass areas adjacent to the Pacific Ocean, the Andes Mountains, the Amazon, and the insular zone, respectively [53]. Ethnically and culturally, the four most representative groups are Mestizos (77.5%), Indigenous (7.7%), Montubios (7.7%), and Afro-Ecuadorians (4.8%). Each has its own customs and historical, social, socioeconomic, and linguistic characteristics, although not all of them are bilingual [54–56]. The Indigenous population has received less European and American influence, Mestizos represent a blend of Aboriginal and European genetic and cultural traits, Montubios are a rural Mestizo population settled in the Costa region, and Afro-Ecuadorians retain cultural elements of their African origins. Given this cultural diversity, there is a compelling need to gather data on executive performance in the preschool population within a context that differs culturally from the more commonly studied settings. Beyond addressing a significant gap in the literature, such data would enable the comparison of preschool executive performance across diverse international contexts. Furthermore, any academic, clinical, and healthcare intervention in Ecuador would lack consistency and objectivity without alignment with scientific evidence. To a certain extent, the data obtained can also be used as a reference for other Latin American countries that share cultural and socioeconomic diversity similar to that of Ecuador.

Adopting the theoretical and scientific framework of the basic structure and development of EFs in preschool-age children, the present study aimed to conduct a cross-sectional analysis of the changes in WM and IC among a broad sample of Ecuadorian preschoolers. Direct measures will be employed, and the analysis will consider age intervals and gender. The existing literature highlights the stability of WM, noting its limited dependence on the modulating effects of the environment, in contrast to IC [19, 24]. This distinction could potentially explain why gender differences are observed in IC but not in WM [44–48]. Given this background, our hypothesis posits that cross-sectional mean scores for WM will show a non-linear progression across preschool age, as reported in studies including American, Asian, and European samples [6, 26, 57]. We anticipate no significant gender differences in WM scores. Regarding IC, our hypothesis is similar to that of WM, except that we anticipate better scores for girls, as identified in American, German, and Asian samples [44, 45].

## Materials and methods

The present study used a cross-sectional design to analyze preschoolers’ executive performance across different age intervals due to the logistical limitations of conducting a longitudinal study.

### Participants

Twenty-one urban preschool centers with different funding sources were included in the study: 11 public, four subsidized, and six private. The sample was composed of 982 Ecuadorian preschoolers (*M* = 53.71; *SD* = 5.71) belonging to a medium socioeconomic stratum (SES: including medium-high, medium, and low-medium) in urban areas. The participants included 496 boys (*M* = 53.77; *SD* = 5.60) and 486 girls (*M* = 53.65; *SD* = 5.83) from nine cities in Ecuador (Guayaquil, Cuenca, Macas, Milagro, Manta, Esmeraldas, San Lorenzo, Otavalo, and Cotacachi), ranging in age from 42 to 65 months Table 1. In addition, 799 regular caregivers participated in the study, ranging in age from 19 to 70 years (*M* = 32.56; *SD* = 8.62). The distribution of caregivers by type of relationship with the preschoolers was as follows: mother (84.4%), father (14.8%), other family members (7.4%). A total of 183 regular caregivers could not be interviewed because of logistical constraints arising from family and work obligations.

**Table 1:** Descriptive statistics of the preschooler sample.

Age (six-month interval)	Total sample/subsample	Boys	Girls	*P-value* *
	*n (M; SD*)	*n (M; SD*)	*n (M; SD*)	
**Total sample/subsample**	982 (53.71; 5.71)	496 (53.77; 5.60)	486 (53.65; 5.83)	.681
**I (42–47 months)**	162 (44.67; 1.71)	76 (44.75; 1.81)	86 (44.60; 1.63)	.590
**II (48–53 months)**	287 (50.61; 1.66)	149 (50.55; 1.61)	138 (50.68; 1.72)	.506
**III (54–59 months)**	353 (56.44; 1.74)	181 (56.46; 1.72)	172 (56.42; 1.77)	.830
**IV (60–65 months)**	180 (61.43; 1.39)	90 (61.33;1.36)	90 (61.53; 1.42)	.335

*p < .05

**p < .001.

### Sampling of preschool centers and participants

Given the resources available for the study, the selection of preschool centers and participants was guided by a strategic approach to ensure a normative sample representative of the majority of the Ecuadorian preschool population located in urban areas [53]. Thus, stratified random sampling was used to select the preschool centers and the participants based on predefined inclusion criteria. In the sampling of preschool centers, consideration was given to the three primary geographic regions (Costa, Sierra, and Amazon) and the three existing types of financing resources (public, subsidized, and private). For the selection of preschoolers, two sublevels of preschool education were considered: sublevel 1 (between approximately 3.5 to 4.5 years), and sublevel 2 (between approximately 4.5 to 5.5 years). Regarding SES, preschoolers belonging to the medium-high SES, medium SES, and low-medium SES levels were grouped into a single sample under the broader category of medium SES. This grouping aimed to cover the normative Ecuadorian population (83.3%), characterized by having essential resources and opportunities for development [53]. Preschool centers catering to high SES (1.9%) and low SES (14.9%) populations were excluded from the sampling due to their limited representation in the country and the cognitive and emotional alterations associated with these environments, respectively [58, 59]. This criterion was implemented to ensure normative and accurate data collection without compromising the specificity and variability of the sample. The result was a diverse set of preschool centers and participants representing different geographic regions of Ecuador, including the western zone (Guayaquil and Milagro), northwest zone (Manta, Esmeraldas, and San Lorenzo), southern zone (Cuenca), northern zone (Cotacachi and Otavalo), and east zone (Macas).

Regarding the SES classification criterion used, SES is a complex multidimensional construct typically assessed through indicators such as household income, parental education, parental occupation, and access to resources, among others [60, 61]. Consequently, SES categorization criteria may vary across heterogeneous international environments due to the methodological diversity and the specific nuances of each social setting [62]. In the present study, an official Ecuadorian instrument was used to comprehensively measure and categorize multidimensional SES [63].

### Inclusion criteria

The inclusion criteria for selecting preschool centers were: 1) being situated in an urban area and 2) having the majority of their preschoolers belong to a medium SES (including medium-high, medium, and medium-low). Preschoolers meeting the following criteria were included as participants in the study: 1) regular attendance in one of the selected centers; 2) not presenting diagnosed or evident learning, neuropsychological, psychiatric, hearing, or language disorders; 3) belonging to a medium SES, according to the classification of the Socioeconomic Level Stratification Survey [64]; 4) having the consent of their legal guardian, and 5) expression of assent to participate in the study. In addition, the usual caregivers were required to be of legal age, reside or have regular contact with the preschooler, and provide a signed Consent Form.

### Instruments and domains

Before finalizing the assessment protocol, a pilot test was conducted on a representative sample of 120 preschoolers from diverse cultural and socioeconomic backgrounds. The purpose of this pilot was to validate assumptions related to verbal comprehension, attention span, correct identification of stimuli, and overall test performance. The results indicated signs of fatigue, suggesting that the assessment protocol should not exceed a duration of 20 minutes. Thus, two direct and specific measures of WM and IC were selected from cognitive assessment batteries with adequate psychometric properties [65, 66]. The chosen protocol focused on measuring visual working memory and motor inhibition, using procedures and stimuli that were easy to identify and execute by children within the specified age range. The decision to employ direct measures was based on the notion that performance tests presented as games [67, 68] offer accurate data on child performance while minimizing potential biases present in the use of indirect measures. The latter often carry the risk of adults overreporting socially desirable behaviors, underestimating contrary behaviors, or encountering difficulties in understanding, interpreting, and describing children’s executive performance from observing their behavior [69]. The protocol’s brief administration and ease of comprehension for Ecuadorian preschoolers facilitated the assessment of a large sample, aligning with one of the methodological aims of this study. Finally, the assessment protocol was designed to accommodate the logistical constraints associated with field work in Ecuadorian preschool centers, considering the daily dynamics of these centers. The protocol also aimed test the accuracy of sampling preschool centers based on SES.

### Assessment protocol for preschoolers

**Zoo Locations (ZL) test of the Wechsler Preschool and Elementary School Scale of Intelligence (WPPSI-IV) [65].** The ZL test was used to measure WM, with a reliability coefficient ranging from .76 to .85 for children aged between 42 and 65 months, where a higher number of hits is taken to indicate better performance. The test comprises 20 items, each involving the examiner arranging cards with images of various animals (previously recognizable by the children) on a template in a pre-established order. After a few seconds, the cards are removed, mixed, and presented to the child, who is then tasked with placing them in their initial locations. Administration of the test takes approximately 6–9 minutes within the age range of the study sample. The test includes various difficulty levels and ends when two consecutive errors are made. This test uses proactive interference, a phenomenon where recalling a previously seen item interferes with the recall of the current item, increasing the task’s difficulty. The test is based on the observe-realize paradigm described by Reznick [70]. It employs a multi-component model that defines two domain-specific storage systems: the phonological loop for verbal information and the visuospatial sketch pad for visual and spatial information [71]. Even in the absence of proactive interference, the simple visual storage required in the ZL involves processing intervention. This aspect measures the ability to manipulate and update information essential to WM [65].

### Statue test of the NEPSY-II: Child Neuropsychological Battery [66]

The Statue, test, a component of the NEPSY-II: Child Neuropsychological Battery, was used to assess motor inhibitory control in response to various external stimuli. It has a test-retest reliability coefficient of .52 for the 3 to 6-year age group. During the test, the child is instructed to maintain a specific position with closed eyes for 75 seconds, while the examiner emits reaction-inducing sounds for 10, 20, 30, and 50 seconds. In this position, the child stands with feet slightly apart, keeping the left arm extended downward, and the right arm flexed at the elbow, perpendicular to the body. The right hand is clenched as if holding a flag, while the left hand may rest on a table or chair for balance. Thus, three possible errors can be recorded, each with a maximum score of 15 points: Total movements, Total eye-opening, and Total words. Higher scores on this test indicate better performance, and administration typically takes approximately 4–6 minutes.

### Survey for usual caregivers

#### Socioeconomic Level Stratification Survey [64]

This survey was employed to determine the SES of each preschool family and to test the accuracy of one of the criteria for sampling centers. This instrument has been validated in the Ecuadorian population. SES is determined by the score obtained on six parameters, ranging from 0 to 1000 points: Housing characteristics (0–236 points), Education level (0–171 points), Economic activity (0–170 points), Possession of goods (0–163 points), Access to technology (0–161 points), and Consumption habits (0–99 points). The sum of the six scores obtained allows for classifying the households into one of five possible levels: Stratum D = low (0–316 points), Stratum C- = medium-low (316.1–535), Stratum C+ = typical medium (535.1–696 points), Stratum B = medium-high (696.1–845 points), and Stratum A = high (845.1–1000 points). The survey takes approximately 10–15 minutes to administer.

### Procedure

Participant recruitment and assessment started on the 28^th^ of October 2019 and concluded on the 15^th^ of March 2023. Data collection was temporarily suspended during COVID-19. Once the permits for the preschool centers were secured, four trained psychologists carried out field work in each chosen center. This team of evaluators received specific training to standardize the assessment procedures and data recording, minimizing the potential for bias. The assessment protocol, administered to both preschoolers and habitual caregivers, took approximately 10–15 minutes. The evaluations and interviews took place in the school classrooms; preschoolers were assessed during class (morning), while the caregivers were interviewed in both the morning and afternoon.

### Ethics statement

The study was approved by the Ethics Committee for Research on Human Beings of the International University of Ecuador (CEU-085-19). Before signing the Consent Form, the legal guardians of the preschoolers received the necessary written and verbal explanations about the study and the participant’s rights. Despite having the informed consent of their legal guardians, only children who verbally consented to participate in the study were assessed. Each participant was assigned a code to guarantee anonymity and data confidentiality. The evaluators and the authors had only the access necessary for data processing. This study followed the ethical principles of the Declaration of Helsinki and the Guidelines for Good Clinical Practice of the European Union.

### Analysis

In coherence with the design of the study and the hypotheses proposed, six-month age intervals were established: I (42–47), II (48–53), III (54–59), and IV (60–65). Once the necessary statistical assumptions had been verified, between-subjects ANOVAs were conducted to explore the effect of age (using age intervals) and gender on the sample’s performance in WM and IC (using mean scores in each age interval). Post-hoc tests were then applied using the Bonferroni method. For these analyses, raw scores were used, a significance level of ≤.05 was established, and partial *η*^2^ was used as the effect size measure. Cohen’s *d* was also calculated to establish the effect size of the mean scores obtained by boys and girls for WM and IC in each age interval. All data was processed using the statistical package SPSS.25 for Windows [72].

## Results

### Sociodemographic and descriptive data

Only 6 of the 982 participants did not complete the tests, mainly due to a lack of collaboration. The number of participants was higher in the second and third age intervals, while a balanced number of boys and girls was observed in each of the four age intervals. No significant differences were found between boys and girls within each age interval Table 1.

### Results for the working memory test

**Fig 1:**
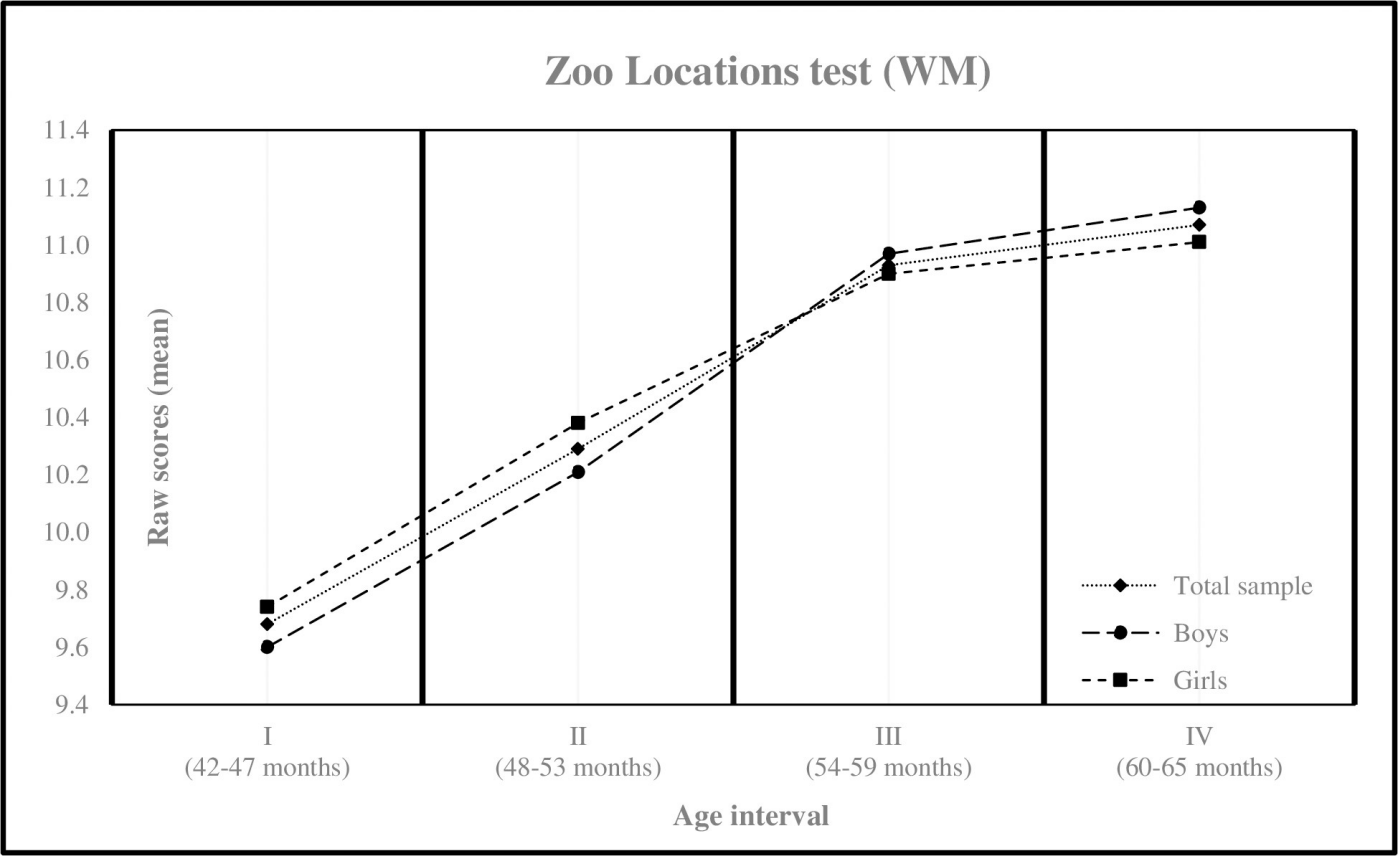
Mean scores for working memory measure by age interval. Zoo Locations = Wechsler Preschool and Elementary School Scale of Intelligence Test, WPPSI-IV [65]; WM = Working Memory.

Table 2 describes the results obtained by age intervals and gender for the WM test. The minimum and maximum values found for each interval were: Interval I (2, 15), Interval II (2, 16), Interval III (8, 17) and Interval IV (7, 17). Moreover, WM differed significantly as a function of age [F (3,971) = 31.046, *p* < .001, *η*^2^ = .088], with children in age intervals III and IV obtaining higher mean scores than those in age intervals I and II Table 3 and Fig 1.

**Table 2:** Means and standard deviations of the scores of preschool children aged between 42 and 65 months for working memory and inhibitory control measures by age-interval and gender.

Variable	Age (six-month interval)	Boys	Girls	*t*	*P-value*	*d*
		*n (M; SD*)	*n (M; SD*)			
**Zoo Locations (WM)**	**I (42–47 months)**	75 (9.60; 1.59)	86 (9.74; 2.02)	-.498	.619	-.08
	**II (48–53 months)**	148 (10.21; 1.88)	138 (10.38; 1.41)	-.884	.377	-.10
	**III (54–59 months)**	180 (10.97; 1.47)	172 (10.90; 1.44)	.496	.620	.05
	**IV (60–65 months)**	90 (11.13; 1.74)	90 (11.01; 1.47)	.138	.612	.07
**Statue (IC)**	**I (42–47 months)**	75 (21.56; 5.75)	85 (23.45; 4.56)	-2.313	**.022** *	-.36
	**II (48–53 months)**	144 (24.43; 4.63)	137 (24.95; 4.78)	-.923	.357	-.11
	**III (54–59 months)**	180 (25.33; 4.24)	171 (26.03; 3.83)	-1.624	.105	.17
	**IV (60–65 months)**	90 (25.71; 4.15)	90 (26.46; 3.71)	-1.268	.206	.19

Zoo Locations = Wechsler Preschool and Elementary School Scale of Intelligence Test, WPPSI-IV [65]; WM = Working Memory; Statue = Children’s Neuropsychological Battery Test, NEPSY-II [66]; IC = Inhibitory Control.

*p < .05

**Table 3:** Results of the two-factor ANOVA for working memory and inhibitory control measures by age-interval and gender.

Test	I (42–47 months)		II (48–53 months)		III (54–59 months)		IV (60–65 months)		*P-value*	Partial *η*^2^	Post-hoc
	Boys	Girls	Boys	Girls	Boys	Girls	Boys	Girls			
	*n* (*M; SD*)	*n* (*M; SD*)	*n* (*M; SD*)	*n* (*M; SD*)	*n* (*M; SD*)	*n* (*M; SD*)	*n* (*M; SD*)	*n* (*M; SD*)			
Zoo Locations (WM)	75 (9,60; 1.59)	86 (9.74; 2.02)	148 (10.21; 1.89)	138 (10.38; 1.41)	180 (10.97; 1.47)	172 (10.90; 1.44)	90 (11.13; 1.74)	90 (11.01; 1.47)	Age**	.088	(I = II) < (III = IV)
Statue (IC)	75 (21.56; 5.75)	85 (23.45; 4.56)	144 (24.43; 4.63)	137 (24.95; 4.78)	180 (25.33; 4.24)	171 (26.03; 3.83)	90 (25.71; 4.15)	90 (26.46; 3.71)	Age**	.069	(I = II) < (III = IV)
									Gender*	.011	G > B

Zoo Locations = Wechsler Preschool and Elementary School Scale of Intelligence Test, WPPSI-IV [65]; WM = Working Memory; Statue = Children’s Neuropsychological Battery Test, NEPSY-II [66] IC = Inhibitory Control. B = Boys; G = Girls.

*p < .05

**p < .001

### Results for the inhibitory control test

**Fig 2:**
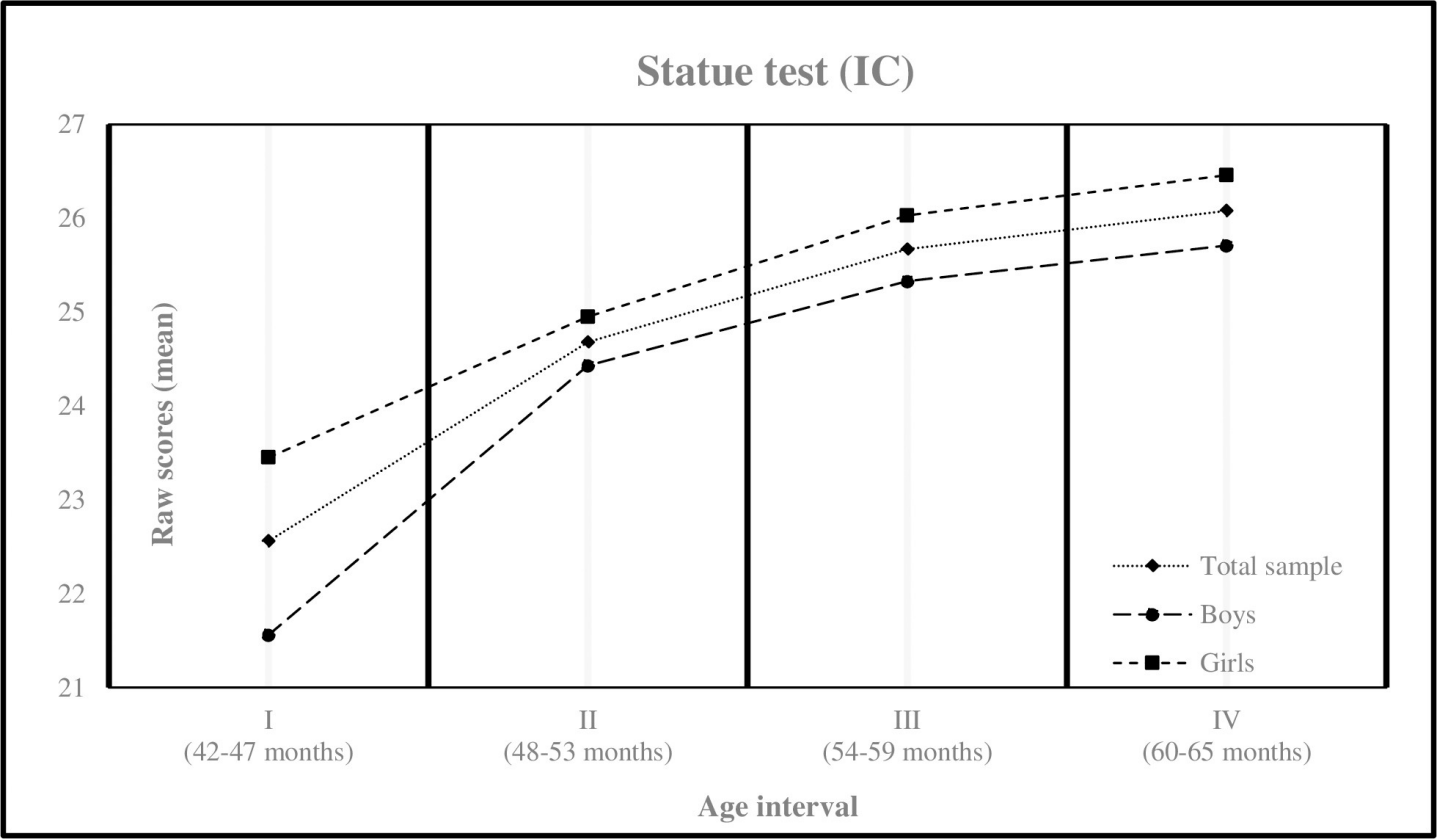
Mean scores in inhibitory control measure by age interval. Statue = Child Neuropsychological Battery Test, NEPSY-II [66]; IC = Inhibitory Control.

Table 2 describes the results obtained by age intervals and gender for the measures of the IC test. The minimum and maximum values found for each interval were: Interval I (5, 30), Interval II (4, 30), Interval III (10, 30) and Interval IV (7, 30). IC also differed significantly as a function of age [F (3, 96) = 23.72, *p* < .001, *η*^2^ = .069], with children in age intervals III and IV obtaining higher mean scores than those in age intervals I and II. Only IC differed significantly according to gender [F (1, 96) = 10.41, *p* .001, *η*^2^ = .011], with girls obtaining higher mean scores than boys in age interval I Table 3 and Fig 2. Consistent with recently reported data, only in age interval I was a medium effect size identified for IC [73].

## Discussion

The present study aimed to conduct a cross-sectional analysis of the changes in WM and IC in a broad sample of Ecuadorian preschoolers using direct measures and considering four 6-month age intervals and gender. Unlike a longitudinal study with the same sample, a cross-sectional study enables comparisons between different groups of the same population studied at various age intervals. Given the methodological constraints inherent in the design of the present study, our findings support the proposed hypotheses. Specifically, we expected that the mean scores obtained on the administered tests would show a non-linear increase according to the age interval studied for both EFs, with gender differences emerging in the case of IC. In fact, the mean scores for the WM test showed an upward trend in each age interval, especially in the first interval, without gender differences. Similarly, the results of the IC test revealed an increment in mean scores across each age interval, with girls outperforming boys Table 3 and Fig 2. Additionally, insignificant effect sizes were found for WM, whereas a medium effect size was observed at the earliest age interval for IC Table 2. These findings warrant a comprehensive discussion exploring both the similarities and differences between our results and the existing literature, particularly in relation to the proposed hypotheses.

As expected, the observed improvement in performance across age intervals for both tests is consistent with the findings reported in the literature Table 3. When viewing the cross-sectional data of the current study as a temporal continuum, trajectories in WM and IC are consistent to those reported in longitudinal studies [8, 22, 26, 57]. Furthermore, these findings coincide with data reported in studies conducted in English-speaking [3, 4, 22, 34], European [5, 6], and Asian contexts [7, 8], including normative samples or those of medium SES. Taken together, these studies suggest that EF performance tends to improve over time during early childhood, showing a hierarchical but non-linear developmental structure [4, 26, 57].

### Performance in working memory

Regarding WM, an evolutionary perspective suggests that it is a relatively stable function, less susceptible to environmental influences compared to other cognitive variables, and tends to stabilize over time [13, 15, 16]. The stability of this function supports the existence of similar findings in such diverse cultural and sociodemographic environments, fostering debate about the possible stability of WM across cultures [7, 74, 75]. Thus, the data from the present study contribute significantly to filling an important gap, while acknowledging the nuances associated with each development context. Consequently, it is essential to explore possible explanations for the observed variability in the data.

From a critical standpoint, it is necessary to consider the accuracy and sensitivity of the employed measures–according to the functional maturity of brain structures–when interpreting results obtained in younger children. For instance, a longitudinal study involving preschoolers highlights that while cognitive development is associated with age, younger children aged between 3 and 4 years show greater variability in their results, suggesting that EF may drive test performance somewhat differently as development progresses [57]. In addition, the authors of this study point out that the variability in scores could be due to the relatively weak psychometric properties of many EFs tests, potentially introducing artifacts into the results.

Another hypothesis worth considering is that the variability in the developmental trajectory of both EFs can be reasonably explained by a combination of maturation and the interaction of the processes involved in early childhood development. In this regard, factors such as the impact of early stimulation within the home and family environment [51, 57], cognitive training in educational settings, motivation, and teaching strategies [29, 39, 76] can play a significant role in shaping children’s performance [17, 75, 77, 78]. Additionally, the heterogeneity and insufficient systematization of teaching methodologies for Ecuadorian preschoolers warrants exhaustive investigation [79–81].

Although no gender differences have been detected for this EF, it is worth highlighting that the present study analyzes executive performance by gender due to the inconsistent data reported in the literature on this subject. In contrast to our findings, previous research with samples from Eastern and Western countries identified better performance of WM skills in boys than girls [47] and vice versa [82]. Considering the stability of WM, any observed gender differences should be examined in the context of various environmental variables such as those already mentioned.

### Performance in inhibitory control

Similar to the WM data, the results of the current study on IC are consistent with those reported in previous studies. However, our data show greater variability compared to the findings from other studies [19, 83, 84]. This variability might be attributed to the characteristics of the sub-processes governing motor response and goal-directed behavior, including mental representations, error awareness, and stimulus-response relationships [4]. Thus, the interaction between emotional regulation, motivation, and arousal with IC could explain its greater variability and sensitivity to environmental factors or learning experiences [19, 83].

An alternative explanation for the observed variability lies in the quality of early stimulation and cognitive training provided to children within the home environment before exposure to the homogenizing influence of the academic context [85–88]. Cross-cultural studies propose that the variability in certain measures could stem from the modulating effect of environmental variables specific to a cultural context. Key environmental variables influencing behavior in early childhood include values [89], family stress and influence [60, 77, 90], parental styles, parenting patterns, expectations or biases of parents [77, 91], quality of early stimulation [78, 92], peer acceptance, teaching-learning strategies [7, 30, 93, 94] and SES [58, 59, 88]. Cross-cultural studies emphasize these environmental variables as contributors, to varying extents, to the variability in early childhood executive performance, while acknowledging the potential impact of methodological weaknesses in data collection, as previously suggested [5, 7, 32, 50, 75, 77, 78]. To enhance the East vs West model, some authors propose conducting intra-country cross-cultural studies, particularly when aiming to identify culture-specific effects on population performance in a given domain [51, 52]. This approach is particularly suited to multicultural countries such as Ecuador.

Concerning gender differences, the existing literature presents conflicting findings. Some studies report no differences [34, 45, 95–97], while others indicate better performance in boys compared to girls [98–100] and vice versa [44, 49, 89, 101]. A possible interpretation, strongly rooted in Ecuadorian culture, is the presence of gender bias in the different training that families provide to their sons and daughters [32, 49, 77]. The cultural environment could shape perceptions of right or wrong, as well as appropriate education and training for each gender, influencing the behavior of boys and girls based on societal expectations [36, 94]. In this regard, some studies emphasize the prevailing belief that boys are naturally more active and impulsive, while girls are deemed more conservative, reflective, and possess better emotional regulation [20, 45, 82, 102, 103]. As preschool centers may not be entirely free of such biases, this trend could be reinforced in this environment. Consequently, the magnitude of this bias is likely to be reflected in the mean scores obtained by boys and girls during assessments [96, 99]. It is evident that this approach to understanding gender differences in early childhood has significant implications for educational management during this crucial developmental stage, impacting the future success of children [1, 104–106]. Ultimately, a comprehensive and consistent data set will provide more precise insights into gender differences in executive performance, including the potential explanatory variables.

### Learning implications

As these are two of the key EFs in early childhood, the observed stability of WM and gender differences in IC among this sample of preschoolers can help to inform the design of several initiatives in learning and academic settings. As a starting point, it is crucial to prioritize the identification of factors contributing to the variability of executive performance in preschoolers. Understanding these factors can provide insights into tailoring educational approaches to the needs of this age group during their initial exposure to academic contexts. Additionally, the consistent gender differences observed in IC deserve equal attention. Furthermore, this analysis should consider the prenatal stage and the first three years of a child’s life within the family context, given the determining impact of these early stages on the cognitive and behavioral development of children [1, 104–106]. Drawing on scientific evidence, it will then be possible to adapt and optimize cognitive training strategies in early childhood, creating learning environments that are free from biases and barriers [14, 27, 33, 102, 107].

### Strengths and limitations

The present study has a number of notable strengths: (a) to the best of our knowledge, this is the first study to adopt this approach in a Latin American preschool population, (b) the study uses a large sample of both preschoolers and regular caregivers, (c) the findings address a critical gap in the literature by examining the developmental trajectory of WM and IC in Latin American preschool population considering age and gender, (d) the study provides the scientific resources to offer a more in-depth analysis of the effect of environmental variables on the development of EF according to age and gender, and (e) although the study has a clear objective, its findings also offer insights that could enrich the development of studies from a cross-cultural perspective.

While the design of a brief protocol facilitated the assessment of a large sample, the inclusion of additional analogous tests could have enriched the dataset, enabling a more comprehensive comparison of the accuracy of the measurements. A significant limitation to consider in future studies, especially in countries with sociocultural diversity, such as Ecuador, is the absence of studying executive performance based on ethnicity or culture. Addressing this limitation and enhancing the generalizability of the results to specific cultural environments would be favored by adopting a more complex methodological approach.

## Conclusion

In summary, viewing the cross-sectional mean scores of the WM and IC tests as a temporal continuum reveals a consistent upward trend in each age interval studied. Girls outperformed boys on the IC test, with statistically significant differences observed in the earliest age interval. Future studies should consider broadening the age range of the sample and applying a more complex methodology. This approach would enable a more comprehensive examination of the impact of gender on executive performance and, if applicable, the environmental variables that contribute to these observed effects.

## Supporting information

S1 Checklist *PLOS ONE* clinical studies checklist. (DOCX)
